# Inhibited hypoxia-inducible factor by intraoperative hyperglycemia increased postoperative delirium of aged patients: A review

**DOI:** 10.1097/MD.0000000000038349

**Published:** 2024-05-31

**Authors:** Yutong Han, Bing Ji, Yulin Leng, Chunguang Xie

**Affiliations:** a Hospital of Chengdu University of Traditional Chinese Medicine, Chengdu, Sichuan Province, People’s Republic of China; b Department of Pain Management, Hospital of Chengdu University of Traditional Chinese Medicine, Chengdu, Sichuan Province, People’s Republic of China; c Traditional Chinese Medicine Regulating Metabolic Diseases Key Laboratory of Sichuan Province, Chengdu, Sichuan Province, People’s Republic of China.

**Keywords:** anesthesia, elderly, HIF, perioperative hyperglycemia, postoperative delirium

## Abstract

The underlying mechanism of postoperative delirium (POD) in elderly people remains unclear. Perioperative hyperglycemia (POHG) is an independent risk indicator for POD, particularly in the elderly. Under cerebral desaturation (hypoxia) during general anesthesia, hypoxia-inducible factor (HIF) is neuroprotective during cerebral hypoxia via diverse pathways, like glucose metabolism and angiogenesis. Hyperglycemia can repress HIF expression and activity. On the other hand, POHG occurred among patients undergoing surgery. For surgical stress, hypothalamic-pituitary-adrenal activation and sympathoadrenal activation may increase endogenous glucose production via gluconeogenesis and glycogenolysis. Thus, under the setting of cerebral hypoxia during general anesthesia, we speculate that POHG prevents HIF-1α levels and function in the brain of aged patients, thus exacerbating the hypoxic response of HIF-1 and potentially contributing to POD. This paper sketches the underlying mechanisms of HIF in POD in elderly patients and offers novel insights into targets for preventing or treating POD in the same way as POHG.

## 1. Introduction

Postoperative delirium (POD) is an acute neurological condition of attention and cognition owing to a variety of factors.^[1]^ The incidence of POD is 11% to 51% in postoperative individuals_,_ and its prevalence increases significantly among the elderly.^[1,2]^ POD is linked with prolonged hospitalization, unfavorable functional recovery, long-term cognitive dysfunction, and increased mortality and costs,^[3]^ especially in elderly surgical patients. Neuroinflammation, metabolic changes in the brain, and impaired neuronal connectivity have been postulated to contribute to POD.^[4]^ However, the exact mechanism underlying POD, especially for the elderly, remains unclear.

More than 20% of the elderly experience cerebral desaturation (hypoxia) after noncardiac abdominal surgery, as an independent risk indicator for POD.^[5–9]^ Hypoxia drives levels of hypoxia-inducible factor-1 (HIF-1), a major transcription factor that modulates hypoxic responses.^[10–15]^ Elevated HIF-1 monitors the level of target genes, which are implicated in erythropoiesis, neuroprotection, and apoptosis and protect tissues from hypoxia.^[11–13]^ Reduced HIF-1-mediated hypoxia responses were observed in aged animal models,^[15]^ which may aggravate tissue impairment induced by hypoxia.^[16]^ Animal research also demonstrates the protection of HIF-1 in ischemic brain.^[14]^ Hippocampal HIF-1α/VEGF axis is the upstream responsible for isoflurane-induced cognitive impairment, providing a potential target for preventing and treating POD.^[16]^

Perioperative hyperglycemia (POHG) occurs among patients undergoing surgery, especially in older adults.^[17–22]^ POD and cognitive impairment are adverse outcomes of intraoperative hyperglycemia (IPHG).^[3–10]^ Acute insulin resistance represents a primary cause of IPHG since surgical stress response facilitates the production of catecholamines and cortisol.^[23,24]^ As an influencing factor of HIF-1,^[17,18]^ hyperglycemia influences HIF-1 levels and functions, which has been widely reported in studies on complications of diabetes.^[19]^ HIF-1-mediated hypoxic adaptation is impaired in diabetes, resulting in cellular dysfunction. Hyperglycemia retards the stability and function of HIF-1 in hypoxic cardiomyocytes,^[13]^ endothelial cells, dermal fibroblasts,^[11,12]^ retinal epithelial cells, and proximal tubular cells.^[15]^

Hence, we speculate that HIF is pivotal for POD in the aged with IPHG. POHG would inhibit HIF-1α expression and the hypoxia response, which would lead to neurological damage and POD in elderly populations. This paper delineates the underlying pathway of HIF in the elderly with POD and how POHG influences HIF-1 expression and function, hoping to find novel targets to restrain POD in the elderly.

## 2. Cerebral hypoxia is pivotal in POD in the aged

Brain hypoxia occurs occasionally in elderly surgical patients. Ehrenfeld et al^[25]^ noted that 6.8% of them went through an intraoperative event of hypoxia, and 3.5% had an event lasting at least 2 minutes. In elderly individuals, a decreased oxygen supply may be caused by many perioperative procedures, like hypoperfusion, acute anemia, fluid overload, hypoventilation, and atelectasis.^[26]^ All surgeries carry a risk of cerebral ischemia due to microthrombosis (especially in the elderly), which may lead to acute and total hypoxia of local brain tissue.^[27,28]^ Anesthetics also trigger cerebral hypoxia, the severity of which depends on the type of anesthetics.^[29]^ A prospective randomized study reported that major abdominal nonvascular surgery led to cerebral desaturation in about 20% of elder populations.^[30]^

Cerebral hypoxia is a potential risk index for POD.^[31–33]^ POD in patients with cardiac surgery and thoracotomy was linked with postoperative cerebral desaturation.^[31,34]^ In addition to heart and lung surgery, surgery in other parts is also related to POD.^[9,30]^ Andrea Casati et al^[30]^ used rSO2 monitoring for anesthesia management in elderly patients with major abdominal surgery, which diminished the potential risk of brain hypoxia and subsequent cognitive decline. The high metabolic rate of neurons^[35]^ requires rapid and precise modulation of cerebral blood flow, making them susceptible to acute and chronic hypoxia. According to a relevant report,^[36]^ lung-protective ventilation is beneficial for preventing POD in elderly people undergoing spinal surgery via a potential mechanism of improved brain oxygen metabolism. This study suggests that improving cerebral oxygen metabolism may be potentially effective for reducing POD in the elderly.

## 3. Role of HIF-1 protects brain from hypoxia

Cerebral hypoxia may cause a series biological reactions, including excitotoxicity, inflammation, oxidative stress, and apoptosis, leading to neurodegeneration. Neurons are very vulnerable to hypoxia, so a compensatory mechanism to hypoxia is crucial for neuronal survival. Hypoxia elevates the levels of multiple genes, including HIF-1, a heterodimer consisting of HIF-1αand HIF-1β subunits. HIF-1α is regulatable while HIF-1β is stable. Under normoxic conditions, HIF-1β is consistently expressed in cells, whereas HIF-1α is rapidly degraded by ubiquitin-dependent proteasome because of oxygen-dependent HIF prolyl hydroxylase (PHD) activation.^[37]^ Insufficient oxygen makes PHD inactive and HIF-1α stable, followed by nuclear translocation to shape heterodimeric complexes with HIF-1β.^[38]^ HIF-1 activates multiple genes that monitor oxygen homeostasis, including genes implicated in oxygen consumption, angiogenesis, erythrocyte production, and mitochondrial metabolism.^[39]^ In brain, responses mentioned above may resist hypoxia/ischemia-induced neurological damage. In fact, the HIF pathway is critical for intrinsic neuroprotection.^[40]^ A protective effect against cerebral ischemia was observed when using desferoxamine pretreatment to upregulate HIF-1α,^[41]^ which was markedly averted by neuron-specific HIF-1 deficiency.^[42]^ Additionally, neuron-specific HIF-1α downregulation aggravated cerebral ischemia damage.^[42,43]^ However, different viewpoints were also reported showing an anti-cell survival effect of HIF-1α.^[44]^ The discrepancy may be owing to differences in type, duration, severity, and stage of the insult.^[45]^ Upon mild conditions, hypoxia stabilizes HIF-1 and elevates levels of downstream protective genes. Nevertheless, under prolonged hypoxia, HIF-1α stabilization associates with increased p53 levels and accelerates the transcriptional activation of pathologic genes.^[46]^ Most surgical patients do not face prolonged hypoxia generally. Therefore, we believe HIF-1 is more likely to be protective in the case of surgery.

## 4. Potential mechanisms of HIF-1 protect brain neurons

Generally, delirium was considered a complex multifactorial causation syndrome. Many mechanisms were proposed to contribute to POD, including inflammation, oxidative stress, metabolic derangements, physiological stressors, electrolyte disorders, and genetic factors.^[1,47,48]^ The neuroprotective effect of HIF-1 may partly be explained by some of the leading mechanisms above.^[49]^

### 4.1. Neuroinflammation

Inflammation was reported as a key pathogenic mechanism of POD.^[50]^ Hypoxia can trigger systemic and central inflammation.^[25]^ Besides, the systemic inflammatory response is common during the operation.^[1]^ Activated microglia, glial cells, and astrocytes generate proinflammatory cytokines and chemokines in central nervous system, which are essential for neuroinflammation. POD patients have markedly more white blood cells, enhanced neutrophil percentage and neutrophil/lymphocyte ratio, and a reduced mean platelet volume.^[51]^ In another research, increased inflammatory cytokines (IL-6, IL-8) levels were found to associate with delirium.^[50]^ In a cell study of nonalcoholic fatty liver disease, HIF-1α silence increases IL-6 secretion and aggravates inflammation in liver cells.^[52]^ HIF-1α, a hypoxia regulatory factor is prominent against acute inflammation of different diseases. Acriflavine hydrochloride, a HIF inhibitor, was reported to elevate pro-inflammatory cytokines and showed an anti-neuroprotective effect.^[53]^ Given the above, HIF-1 reduces the level of inflammatory cytokines through multiple pathways and plays a neuroprotective role.

### 4.2. Oxidative stress

It is widely reported that abnormally activated oxidative stress is related to the potential mechanism of POD.^[54,55]^ Increased oxidative stress and disordered serotonergic neurotransmitters were seen in delirium patients.^[56]^ Several studies report that oxidative stress was affected by HIF-1α via multiple mechanisms. For instance, HIF-1αprevents oxidative stress through a mitochondria pathway.^[57]^ Mitochondrial ferritin prevents cerebral cell death induced by hypoxia by binding to uncommitted iron and reducing redox damage.^[58]^ Besides, it may prevent redox damage by upregulation of mitochondrial ferritin.^[58]^ FG4592 (Roxadustat), a kind of HIF stabilizer, can promote levels of antioxidant superoxide dismutase 2.^[59]^ A recent study of cerebral ischemia/reperfusion injury indicated that the HIF-1 pathway may enhance anti-oxidative stress process, playing a role of self-protection.^[60]^

## 5. Hyperglycemia affects HIF-1 activity in elderly patients

**Figure 1.**
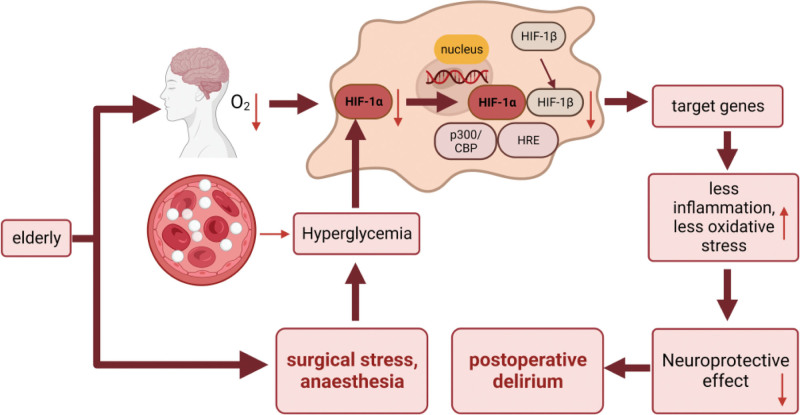
A hypothetical model for the relation between inhibited HIF by IPHG and POD in the elderly. (1) Under cerebral desaturation (hypoxia) during general anesthesia, hypoxia drives HIF-1 expression, which monitors the hypoxic response. (2) IPHG curbed HIF-1α synthesis and activation in aged patients, which led to a decreased neuroprotective effect. HIF-1 = hypoxia-inducible factor-1, IPHG = intraoperative hyperglycemia, POD = postoperative delirium.

As an influencing factor of HIF-1,^[17,18]^ hyperglycemia influences HIF-1 levels and functions, which has been widely reported in studies on complications of diabetes,^[19]^ especially in the elderly. We proposed a hypothetical model for the pathophysiological relation between inhibited HIF by IPHG and POD in the elderly (Fig. 1).

Studies have shown that in the brain of the elderly, hypoxia response mediated by HIF-1α is weakened or even absent in some cases.^[15,61,62]^ Under normal oxygen saturation, VEGF levels were lower in carotid bodies of old rats, and the response to 12% O_2_ for 12 days was weaker.^[15]^ The aged animal model revealed a lack of cortical vessels and insufficiently expressed HIF-controlled proteins (such as erythropoietin, Inos, and heme oxygenase-1) after hypoxia, along with less mitochondria in smaller size within carotid bodies.^[15,61]^ In addition, PHD which modulates HIF-α levels also lost its reactivity during aging.^[63]^

Hyperglycemia was reported to affect HIF-1 levels and functions.^[17,18]^ A study in vitro revealed a potential detrimental effect of hyperglycemia on tissue adaptation to hypoxia through down-regulation of HIF-1,^[64]^ suggesting a new mechanism for hypoxia-induced cell damage. The appearance which hyperglycemia influences HIF-1 levels and functions has been widely reported in complications of diabetes,^[19]^ which may lead to cellular injury in different organs (such as myocardium, retina). For instance, hyperglycemia hinders VEGF response to hypoxia by repressing the HIF pathway in immortalized rat proximal tubular cells.^[65]^ Several papers have reported that blockade of HIF-1 pathway attenuates HIF-1 activation in diabetic tissues under hypoxia. HIF-1 stability and transcriptional activation are impaired in wounds,^[64,66]^ kidney,^[67]^ and heart^[68,69]^ of diabetic patients and animal models. Hyperglycemia blocks HIF-1 levels and functions under hypoxia in cardiomyocytes,^[68]^ dermal fibroblasts and endothelial cells,^[64,66]^ retinal epithelial cells^,[70]^ and proximal tubular cells.^[65]^ Similar changes were also found in central nervous system, HIF-1 and its downstream VEGF were notably less in the brain of diabetic rats than in normal rats, which presented worsened neurological deficits.^[71]^ Accumulating evidence^[66,69,72–74]^ suggests that impaired hypoxic responses because of HIF-1 pathway dysregulation are vital pathogenic factors of diabetic complications. Older age is also a risk index for POHG.^[75]^ This may be explained by the decrease of insulin sensitivity related to aging, owing to mitochondrial dysfunction, enhanced abdominal fat mass, lower levels of insulin-like growth factor 1 and dehydroepiandrosterone hormone, and oxidative stress and inflammation.^[76]^

The study had shown that POD was related to IPH.^[3–10]^ POHG occurs among patients undergoing surgery, especially in older adults.^[20–22,75]^ About 20% to 40% of patients receiving general surgery experienced POHG.^[20,76,77]^ Among them, 12% to 30% of patients with POHG were free of diabetes prior to surgery,^[78]^ which is known as “stress hyperglycemia.”^[79]^ During physiologic stress, hypothalamic-pituitary-adrenal activation and sympathoadrenal activation raise endogenous glucose production through gluconeogenesis and glycogenolysis.^[80]^ In normal settings, glucose stability is strictly regulated by insulin-mediated peripheral glucose uptake and inhibition of liver glucose production. However, under surgery conditions, temporary insulin resistance and repressed insulin release usually result in hyperglycemia.^[81]^ Hyperglycemia may last several days after surgery and have negative impacts on postoperative outcomes.^[82]^

## 6. Conclusion

Up to now, the mechanism underlying POD remains unclear. From the above studies, under the setting of cerebral desaturation (hypoxia) during general anesthesia, we speculate that POHG inhibited HIF-1α levels and function in the brain of aged patients and thus exacerbated the hypoxic response of HIF-1and contribute to POD. This may hint at a novel mechanism of neuronal injury due to POHG and POD. To prove this, further research needs to be done.

## Author contributions

**Conceptualization:** Yutong Han, Bing Ji.

**Project administration:** Chunguang Xie.

**Supervision:** Yulin Leng, Chunguang Xie.

**Writing – original draft:** Yutong Han.

**Writing – review & editing:** Bing Ji, Yulin Leng, Chunguang Xie.
